# Radiotherapie oder inguinofemorale Lymphadenektomie bei Vulvakarzinompatientinnen mit Mikrometastasen im Wächterlymphknoten

**DOI:** 10.1007/s00066-022-01922-5

**Published:** 2022-04-12

**Authors:** Simone Marnitz, Anne-Sophie Bereuter

**Affiliations:** Klinik für Radioonkologie, CyberKnife und Strahlentherapie, Univ.-Klinik zu Köln, Kerpener Str. 62, 50937 Köln, Deutschland

## Begründung/Ziele der Arbeit

Vor dem Hintergrund der hohen Rate therapiebedingter Morbidität, vor allem Lymphödeme, Wundheilungsstörungen und Infektionen, hat die *Groningen International Study on Sentinel Nodes in Vulva Cancer* (GROINSS-V II) in der hier diskutierten Studie untersucht, ob die Strahlentherapie der Leisten eine onkologisch sichere Alternative zur inguinofemoralen Lymphonodektomie (LNE) bei Patienten mit Vulvakarzinomen ist, die in dem/den Sentinel-Lymphknoten Lymphknotenmetastasen aufweisen.

## Methoden und Patientengut

Bei der vorliegenden Studie handelt es sich um eine nichtrandomisierte, prospektive, multizentrische Single-arm-Phase-II-Studie. Eingeschlossen wurden Patientinnen mit einem frühen Vulvakarzinom mit einem Durchmesser von bis zu 4 cm ohne klinische oder bildgebende Zeichen einer Lymphknotenmetastasierung. Sie wurden primär einer lokalen Exzision und einer Sentinel-Lymphknoten-Biopsie unterzogen. Im Falle eines positiven „sentinel node“ (SN) wurde, unabhängig von der Metastasengröße, eine inguinale Radiotherapie mit 50 Gy indiziert. Auf eine LNE, die bisher leitlinienbasierte Therapieempfehlung bei positivem SN wäre, wurde dabei verzichtet. Der primäre Endpunkt war die isolierte Lymphknotenrezidivrate in der ipsilateralen Leiste nach 24 Monaten. Strenge Kriterien wurden bezüglich der Leistenlymphknotenrezidive gewählt vor dem Hintergrund, dass Rezidive in den Leistenlymphknoten bei Vulvakarzinomen immer einen fatalen Ausgang nehmen.

## Ergebnisse

Zwischen 2005 und 2016 konnten 1535 geeignete Patientinnen registriert werden. Der SN war positiv bei 322 Patientinnen (21 %). Viereinhalb Jahre nach Beginn der Studie, im Juni 2010, waren die Abbruchkriterien erfüllt, da von den 91 sentinel-positiven Patientinnen 10 ein isoliertes Lymphknotenrezidiv entwickelt hatten. In der Analyse zeigte sich, dass 9 dieser 10 Patientinnen eine Lymphknotenmetastase im SN von > 2 mm oder ein extrakapsuläres Wachstum aufwiesen. Daraufhin wurde das Protokoll geändert, sodass fortan Patientinnen mit einer Lymphknotenmetastase > 2 mm eine systematische LNE (gültiger „standard of care“) erhielten. Bei Patientinnen mit Mikrometastasen von maximal 2 mm Größe wurde aber weiterhin eine alleinige Strahlentherapie durchgeführt. Von den 160 Patientinnen mit SN-Mikrometastasen erhielten also insgesamt 126 eine inguinale Radiotherapie; von ihnen entwickelten 1,6 % nach 2 Jahren ein inguinofemorales Rezidiv.

Unter den 162 in die Studie eingeschlossenen Patientinnen mit SN-Makrometastasen betrug die isolierte Rezidivrate in den Leisten nach 2 Jahren inakzeptable 22 % (!!), wenn die Patientinnen nach SN lediglich eine Strahlentherapie erhalten hatten. Die Rezidive betrugen nur 6,9 %, wenn eine inguinofemorale LNE +/− Strahlentherapie erfolgt war. Die Toxizität war wie erwartet günstiger bei Patientinnen, die keine inguinofemorale LNE erhalten hatten.

## Schlussfolgerung der Autoren

Bei positiven SN in der Leiste und einer Metastasengröße > 2 mm (= Makrometastase) bleibt die inguinofemorale LNE weiterhin der therapeutische Standard. Bei Mikrometastasen bzw. einer Metastasengröße bis 2 mm kann unter Verweis auf die vorliegende Studie auf eine inguinofemorale LNE verzichtet werden, sofern eine Strahlentherapie mit 50 Gy durchgeführt werden soll.

## Kommentar

Der Verzicht auf die LNE vor dem Hintergrund einer deutlichen therapiebedingten Toxizität ist seit 30 Jahren ein Thema in der gynäkologischen Onkologie. Historisch betrachtet gibt es dazu zwei randomisierte Studien, eine von *Stehman et al. *1992 publiziert [1], die den Stellenwert der inguinalen Lymphonodektomie gegenüber der Strahlentherapie bei Patientinnen mit Vulvakarzinomen T1–3 bei klinisch* unauffälligen* Leisten (cN0) untersuchte. Die eine Gruppe erhielt dabei keine inguinale LNE, dafür aber eine Strahlentherapie, die sicher zum damaligen Zeitpunkt in Technik und Dosierung mit dem heutigen Standard nicht zu vergleichen ist. Diese Patientinnen entwickelten zu 18,5 % Leistenrezidive. Die andere Gruppe erhielt eine Lymphonodektomie und eine Strahlentherapie in Abhängigkeit vom Befallsmuster; diese Patientinnen entwickelten keine (0 %) Leistenrezidive. Damit war bei radiologisch und klinisch unauffälligen Leisten die Strahlentherapie als gedachte Alternative zur Lymphonodektomie vom Tisch.

Die zweite Studie [2, 3] beschäftigte sich mit der Frage, ob bei Patientinnen mit nachgewiesenen *1–3 positiven Lymphknoten* und inguinofemoraler LNE auf eine Strahlentherapie verzichtet werden kann. Es wurden 55 Patientinnen in der einen Gruppe ohne Strahlentherapie behandelt mit dem Ergebnis, dass 13 Rezidive auftraten. Die andere Gruppe enthielt 53 Patientinnen, die nach Lymphonodektomie auch eine Strahlentherapie des Beckens und der Leiste erhielten mit nur einem einzigen Leistenrezidiv. Somit war für 30 Jahre in Stein gemeißelt, dass beim Nachweis von Leistenlymphknotenmetastasen eine Radiatio indiziert ist. Einziger Diskussionspunkt war und ist die Anzahl der befallenen LK, also der Cut-off für den Einsatz der Strahlentherapie [4].

Mit der Sentinel-Technik steht uns heute eine Methode zur Verfügung, die aufgrund des geforderten Ultrastagings und der zwingend geforderten begleitenden Immunhistochemie eine sehr hohe Detektionsrate bietet. Vor diesem Hintergrund gingen die Autoren mit ihrer Studie noch einmal der Frage nach, ob nach positiver Sentinel-Detektion im besten Fall die Entfernung des einen und ausschließlich befallenen Lymphknotens auf eine LNE verzichtet werden kann und so den Patientinnen lebenslang die Morbidität der LNE erspart.

Die hier diskutierte Arbeit gibt uns einen Einblick in die gravierenden Nebenwirkungen. So zeigt die Abb. 4 im Paper nach 6 Monaten die Ödemrate nach SN-Biopsie mit 5 und 4 % nach 12 Monaten sowie eine Rate rezidivierender Erysipele von 6,6 %. Die Sentinel-node-Biopsie, gefolgt von Strahlentherapie, führt fast zu einer Verdreifachung der Ödemrate nach 6 Monaten mit 16,4 und 11 % nach 12 Monaten sowie zu einer Verdopplung der Erysipelrate auf 13,6 %. Dramatisch schlechter werden die Ergebnisse noch einmal durch den Einsatz von SN, gefolgt von LNE +/− Strahlentherapie, mit einer Ödemrate von 32 bzw. 22,9 % und einer Erysipelrate von 16,6 %. Diese Raten sind gravierend für die Patientinnen bezüglich ihrer Lebensqualität, aber auch bedrohlich unter Umständen bei rezidivierenden Septikämien durch Erysipele. Vor dem Hintergrund ist die Reduktion therapiebedingter Morbidität für die Patientinnen bezüglich ihrer Lebensqualität und des Körperbilds von entscheidender Bedeutung, aber auch bezüglich der gesundheitsökonomischen Folgekosten der Therapienebenwirkungen. Insofern ist die Studie vom Ansatz her zu begrüßen. Sie zeigt aber auch ein weiteres Mal, dass die Sentinel-Biopsie keine valide Alternative darstellt, die vergleichbar wäre mit dem onkologischen Effekt einer inguinofemoralen LNE. Die biologische Erklärung dafür ist in den möglicherweise metastatischen, aber nicht detektierten Non-sentinel-Lymphknoten zu suchen, wo gegebenenfalls Mikrometastasen weiterhin unerkannt bleiben.

## Fazit

Somit schlussfolgern die Autoren nachvollziehbar, dass die onkologische Sicherheit des Verzichts auf eine LNE nur nach einer Sentinel-node-Biopsie plus Strahlentherapie bei Mikrometastasen von maximal 2 mm Größe gegeben ist. Hier beträgt das isolierte Risiko für Leistenrezidive <2 %. Bei Lymphknotenmetastasen von über 2 mm Größe bleibt die LNE State of the Art. Unbeantwortet bleibt allerdings die Frage, ob ggf. mit einer höheren Bestrahlungsdosis möglicherweise eine bessere lokale Tumorkontrolle erzielt werden kann und ob der Einsatz der simultanen Chemotherapie zusätzlich zur Strahlentherapie auch hier einen therapeutischen Gewinn bewirkt. Dies sollte Gegenstand weiterer klinischer Studien sein.

*Simone Marnitz und Anne-Sophie Bereuter, Köln*
